# Cutting the root: the next generation of T cells engagers against cancer stem cells to overcome drug resistance in triple-negative breast cancer

**DOI:** 10.20892/j.issn.2095-3941.2022.0745

**Published:** 2023-03-24

**Authors:** Jiali Zhang, Bin Liu, Minchao Lyu, Yourong Duan

**Affiliations:** State Key Laboratory of Oncogenes and Related Genes, Shanghai Cancer Institute, Renji Hospital, School of Medicine, Shanghai Jiao Tong University, Shanghai 200032, China

Triple-negative breast cancer (TNBC) is the most difficult type of breast cancer to treat. TNBC is defined by the lack of expression of three receptors: estrogen receptor (ER); progesterone receptor (PR); and human epidermal growth factor receptor 2 (HER2). Chemotherapy is currently first-line treatment for TNBC; however, due to the high heterogeneity of TNBC, most patients eventually develop chemotherapy resistance, which is associated with a poor prognosis^1,2^. Emerging immune checkpoint blockade (ICB) therapies have been shown to have promising therapeutic efficacy in treating solid tumors. A phase III clinical trial reported that the combination of ICB and chemotherapy lengthened progression-free survival in patients with metastatic PD-L1+ TNBC^3^; however, most patients had primary resistance or acquired resistance to ICB. Thus, the intrinsic mechanisms underlying ICB resistance are still under investigation^4^.

## Cancer stem cells (CSCs) are selectively enriched in TNBC upon treatment and contribute to therapy resistance and tumor recrudescence

CSCs are sub-population of tumor cells with self-renewal and high tumorigenicity properties, and are considered to be the main reason for tumor heterogeneity. Studies have indicated that in many solid tumors, including TNBC, CSCs are responsible for tumor metastases and resistance to chemotherapy, ultimately leading to tumor relapse. Moreover, non-CSC tumor cells can adapt to changes through cellular reprogramming, such as the epithelial-to-mesenchymal transition (EMT) and regenerating CSCs^5^. Chemotherapy not only has a limited killing effect on CSCs TNBC, but also promotes the generation of drug-resistant CSCs.

CSCs evade immune surveillance by actively interacting with immune cells in the tumor microenvironment (TME) by secreting soluble mediators and receptor/ligand direct interactions to promote the immunosuppressive TME and tumor stemness^6,7^. CSCs secrete soluble mediators to modulate the immune cells in the TME: CCL2 recruit macrophages, MDSCs and Treg cells; CSF1 polarize tumor-associated macrophages (TAMs); TGFβ promote immunosuppressive myeloid cell activity, T-cell and NK cell inhibition, Treg cell polarization and support the CSC phenotype. CSCs also upregulate PD-L1 to inhibit T and NK cell activity and promote EMT in patients with TNBC^8^. CSCs also downregulate the surface expression of adhesion molecules, such as CD103 ligand and E-cadherin, through EMT to evade CD8+CD103+ memory T lymphocytes^9^. A recent study reported that ICB therapy selects enriched CSCs with reduced antigen processing/presentation machinery (APM) to facilitate ICB resistance in patients with TNBC^10^. TNBC tissues and cell lines are characterized by CD44+CD24− and/or increased aldehyde dehydrogenase (ALDH) activity compared to other breast cancer subtypes. Chemotherapy and ICB currently used in clinical practice enrich tumor stem cells, thereby causing drug resistance and recurrence^11^. Because CSCs contribute to intra-tumoral heterogeneity and promote therapy resistance, successful treatment against CSCs is required to eradicate tumors; however, due to the similarity between CSCs and stem cells, avoiding on-target/off-CSC toxicity remains challenging.

## T-cell engagers (TCEs) strategy has the potential to specifically kill poorly immunogenic CSCs

**Figure 1:**
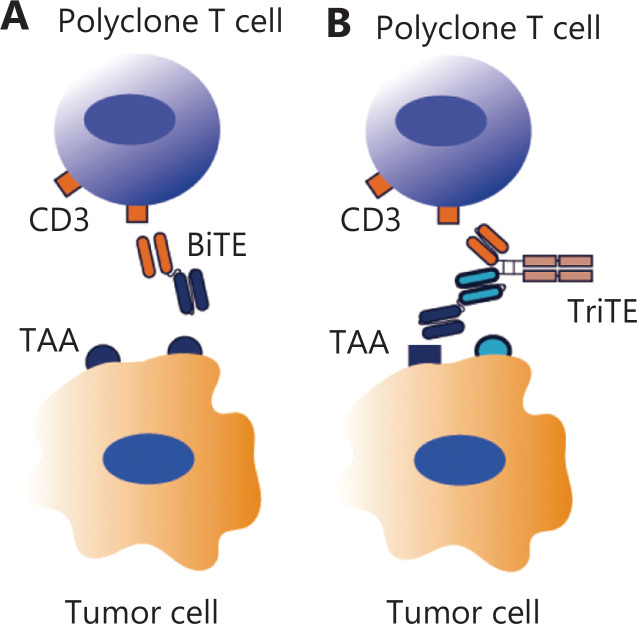
Illustration of bi-specific TCE (BiTE) and tri-specific TCE (TriTE). (A) BiTE binds to tumor-associated antigens (TAAs) on tumor cells and CD3 on T cells to redirect polyclonal T cells to kill TAA-positive tumor cells. (B) TriTE incorporates a T cell binding motif and two TAA binding motifs to overcome TAA heterogeneity or TAA loss during treatment.

TCEs bind to tumor-associated antigens (TAAs) on tumor cells and CD3 on T cells to redirect polyclonal T cells to eliminate tumor cells independent of the antigen specificity of the T cell receptors (TCRs), co-stimulation receptor activation, or cytokine stimulation. As shown in **Figure 1A**, bi-specific T cell engager antibodies (BiTEs) are typical TCEs, which have achieved great success in hematomas; the curative effect is comparable to chimeric antigen receptor (CAR)-T cell therapy. TCE therapy has also shown promise in redirecting polyclonal T cells to kill poorly immunogenic CSCs^12^; however, the function of TCEs relies exclusively on TAAs expressed by tumor cells. Tumor cells may have primary or acquired resistance to TCEs due to TAA expression heterogeneity or loss. Tri-specific TCEs, the next generation of TCEs, incorporate a T cell binding motif and two TAA binding motifs (**Figure 1B**). Tri-specific TCEs target more than one TAA on tumor cells to overcome TAA heterogeneity or loss during treatment. Tri-specific TCEs targeting CD3, epidermal growth factor receptor (EGFR), and epithelial cellular adhesion molecule (EpCAM) were developed to specifically kill EGFR and/or EpCAM-expressing colorectal cancer cells^13^. This tri-specific TCE has enhanced tumor targeting ability and better therapeutic efficacy than the corresponding bi-specific controls. Tri-specific TCEs simultaneously engage TAAs to promote T cell binding and subsequent tumor lysis; however, tri-specific TCEs may also enable T cells to kill normal cells upon binding to TAAs, which are expressed on normal cells. This on-target/off-tumor toxicity significantly hinders TCE applications. The trophoblast cell-surface antigen 2 (Trop2), carcinoembryonic antigen cell adhesion molecule 5 (CEACAM5), erythropoietin-producing hepatocellular carcinoma receptor A10 (EphA10), EpCAM, EGFR, and mesothelin are potential TAAs for TNBC; however, it is difficult to identify TAAs that are specifically expressed on tumor cells, especially CSCs. The goal of the next generation of bi-specific antibodies is to improve tumor-targeting specificity of TCEs to increase therapeutic efficacy, while minimizing on-target/off-tumor toxicity.

## TME-specific activated TCEs are expected to resolve the problem of on-target/off-tumor toxicity and expand the selection of TAAs

**Figure 2:**
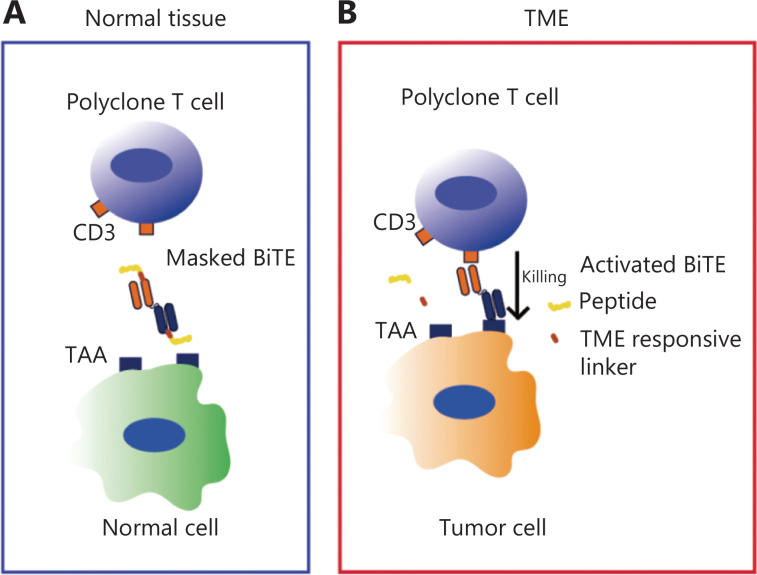
Illustration of the tumor-specifically activated TCEs *in vivo*. (A) In normal tissues, peptide-masked TCEs are basically intact. The masked peptide prevents the antibody from binding to the target due to the shielding, thus preventing T cell activation and avoiding side effects on healthy tissues. (B) When the masked TCEs reach the TME, the tumor-related protease cleaves the substrate linker, thereby releasing the masked peptide, facilitating TCE selective binding to the target antigen in the tumor tissue.

Novel TCEs that are specifically activated in the TME have been developed using protease response peptide masking technology to avoid on-target/off-tumor toxicity. The peptide is connected to the antigen recognition region of the TCEs by a TME-associated protease cleavable linker. The peptide acts as a space shield to mask the antigen recognition ability of TCEs. Peptide-masked TCEs enter body tissues *via* circulating blood. In healthy tissues, peptide-masked TCEs are largely intact. The masked peptide prevents antibody from binding to the target due to the shielding, preventing T cells activation and avoiding side effects on healthy tissues. In addition, masked TCEs have a long half-life and continue to circulate in the blood. The masked TCEs are inactive until activated by TME-associated proteases. The tumor-related protease cleaves the substrate linker when the masked TCEs reach the TME, thereby releasing the masked peptide and facilitating TCE selective bind to the target antigen in the tumor tissue. The activated TCEbind the antigen and CD3 on the tumor surface, thus activating T cells to kill the tumor. One study developed an anti-idiotypic anti-CD3 single-chain variable fragment (scFv)-masked TCE to block the anti-CD3 fragment antigen-binding (Fab) region, which is intact in the systemic circulation and healthy tissues, thus the anti-CD3 Fab region cannot redirect T cells to kill TAA-expressing cells^14^. The tumor-specific protease-cleavable linker between the mask and anti-CD3 Fab region is preferentially unmasked and the activated TCE in the TME specifically kills TAA-expressing cells (**Figure 2**). Thus, the masked TCE strategy enhances the specificity and safety of TCE therapy and expands the selectable TAAs.

## Immunosuppressive TME of solid tumors contributes to the primary and acquired resistance of TCEs

Among solid tumors, especially the ‘immunologically cold’ TNBC, the immunosuppressive TME and the lack of sufficient tumor-infiltrating lymphocytes (TILs) leads to TCE primary or acquired resistance. The immunosuppressive immune cells (MDSCs, TAMs, and Treg cells) in the TME inhibit T cell recruitment and activation. High levels of PD-L1 expression in TNBC inhibit T cell activation and promote T cell exhaustion^15^. Conventional TCE therapy can hardly reverse an immunosuppressive TME. Thus, overcoming an immunosuppressive TME within TNBC is the next challenge for successful TCE therapy. Moreover, the ultimate goal of immunotherapy is to generate long-lasting antitumor reactions that require the collaboration of innate and adaptive immune cells. TCEs redirect T cells to kill TAA-expressing tumor cells, but has a limited effect on innate immune cells. Therefore, a better therapeutic strategy is needed to reverse the immunosuppressive microenvironment and activate innate immune cells to cooperate with TCE therapy to involve the entire immune circulation against cancer; however, the combination of multiple immunomodulators often leads to increased toxicity. There is currently a lack of targeted immunotherapy for TNBC CSCs.

Next-generation TCEs should be designed to have additional functionalities to overcome an immunosuppressive TME, including blocking the immune checkpoint, recruiting T cells and dendritic cells (DCs) to the TNBC TME, and activating innate and adaptive immune cells. A TME responsive flexible platform/delivery system integrates multiple functionalities to enable the delivery of immunomodulatory therapeutics, such as ICB immunoagonist,and TCEs in the TME that reverse the immunosuppressive microenvironment to promote TCE activity is needed, which would be more precise, potent, and safer than the systemic administration of individual immunomodulators and TCEs.

## Activation of type I interferon (IFN) signaling to reverse the immunosuppressive TME and reverse CSCs

CSCs, which are enriched in TNBC and contribute to intratumor heterogeneity, are characterized by an increased expression of multiple immunosuppressive pathways^16^. In addition, chemotherapy selectively enriches CSCs with repressed type I IFN signaling, while activation of type I IFN intrinsically reduces CSC properties and reverses EMT^17^. Moreover, IFN signaling increases the APM of tumor cells, making tumor cells visible to the immune system. Type I IFN also activates innate and adaptive immune cells in the TME, including increased DC maturation, antigen presentation, cross-priming, and recruiting additional T cells to facilitate the generation of long-lasting antitumor immune memory reactions. Therefore, activating the IFN pathway inhibits CSCs and reverses the immunosuppressive TME. Recent findings demonstrated that delivery of a stimulator of IFN gene (STING) agonists in tumor tissues upregulate the transcription of genes that encode type I IFNs, and proinflammatory cytokines and chemokines, which leads to the accumulation and infiltration of CD8+ T-cells and DC maturation, while repression of STINGs in TNBC promotes immune evasion and resistance to ICB^18^. Moreover, preclinical studies with STING agonists have shown promising antitumor efficacy. Thus, STING agonists may act synergistically with TCEs to better inhibit CSCs, reverse the immunosuppressive TME, and generate antitumor immune memory reactions. The metabolic instability, limited tumor targeting ability, and systemic toxicity of STING agonists hinder clinical application^19^. Both TCEs and STING agonists have on-target/off-tumor toxicity upon systemic administration. Thus, the combined administration of STING agonists and TCEs may cause unacceptable toxicity.

## Nano-delivery platform integrates multiple functionalities to achieve TCE and STING agonist combination therapy

Taking advantage of nanotechnology, we adopted a modular design of a multifunctionality platform using a poly(amidoamine) [PAMAM] dendrimer with an ultrasmall size (∼4 nm), adjustable positive charge, and many cavities as substrates to create a TME-responsive Methoxy Poly(ethylene glycol) (mPEG) masked CD44×PD-L1×CD3 tri-specific T-cell nanoengager (PmTriTNE) loaded with a negatively-charged STING agonist [c-di-AMP (CDA)] to overcome the immunotherapeutic resistance of TNBC^20^. PAMAM works as a flexible platform to integrate multiple functionalities without sophisticated protein engineering compared to tri-specific antibodies. We used mPEG with a weakly acidic responsive linker as a mask to shield TriTNEs, which were largely intact in systemic circulation but preferentially unmask TriTNEs in the weakly acidic TME to minimize off-target toxicity while expanding the targets for TAA selection. Taking advantage of the PEG mask, CD44 and PD-L1 were chosen as TAAs. These proteins have been reported to be overexpressed on TNBC cells, and both are important for CSC maintenance and immune escape. We chose an anti-PD-L1 antibody to target and block PD-L1. The oligosaccharide Hyaluronic acid (HA) has modest affinity for CD44 and preferentially targets TNBC cells with high CD44 expression rather than CD44-expressing normal cells. This dual TAA-targeting strategy improves selectivity and binding affinity to tumor cells and preferably overcomes TAA heterogeneity and PD-L1-mediated immune escape.

We determined that the TME-responsive PEG mask provides a half-life extension and enables the selection of TAAs that are also expressed in normal cells^20^. TriTNE targets CD44 and PD-L1, which are enriched in both non-CSCs and CSCs of TNBC (especially in CSCs) to avoid TAA heterogeneity. CDA activates the CSC IFN pathway to intrinsically increase CSC immunogenicity and reverse CSC properties. CDA also stimulates DC maturation and tumor antigen cross-priming, subsequently recruiting CD8+ T and NK cells. TriTNEs block PD-L1 on tumor cells and enables CD8+ T cells to directly kill tumor cells. Then, dead cancer cells release damage-associated molecular pattern(DAMP) and tumor antigens to recruit and activate innate and adaptive immune cells, thus achieving multilayer control of non-CSCs and CSCs in TNBC and generate long-lasting antitumor immunity. PmTriTNE@CDA eliminated TNBC in cell line-derived xenograft (CDX) and patient-derived xenograft (PDX) mice, suggesting a new strategy for overcoming immunotherapy resistance in TNBC. PmTriTNE@CDA was designed to integrate various immune functions into one nanoplatform, thereby enhancing efficacy without compromising safety. Because CSCs contribute to inter-tumoral heterogeneity and immune escape across multiple solid tumor types, this proof-of-concept nanoplatform could broaden the reach of the TCE strategy to many types of cancer and pave the way for new CSC-targeted therapies and rational combination approaches.
